# A Dynamic Selection Method for Reference Electrode in SSVEP-Based BCI

**DOI:** 10.1371/journal.pone.0104248

**Published:** 2014-08-06

**Authors:** Zhenghua Wu, Sheng Su

**Affiliations:** 1 School of Computer Science and Engineering, University of Electronic Science and Technology of China, ChengDu, China; 2 Key Laboratory for NeuroInformation of Ministry of Education, School of Life Science and Technology, University of Electronic Science and Technology of China, ChengDu, China; University of Minnesota, United States of America

## Abstract

In SSVEP-based Brain-Computer Interface (BCI), it is very important to get an evoked EEG with a high signal to noise ratio (SNR). The SNR of SSVEP is fundamentally related to the characteristics of stimulus, such as its intensity and frequency, and it is also related to both the reference electrode and the active electrode. In the past, with SSVEP-based BCI, often the potential at ‘Cz’, the average potential at all electrodes or the average mastoid potential, were statically selected as the reference. In conjunction, a certain electrode in the occipital area was statically selected as the active electrode for all stimuli. This work proposed a dynamic selection method for the reference electrode, in which all electrodes can be looked upon as active electrodes, while an electrode which can result in the maximum sum relative-power of a specific frequency SSVEP can be confirmed dynamically and considered as the optimum reference electrode for that specific frequency stimulus. Comparing this dynamic selection method with previous methods, in which ‘Cz’, the average potential at all electrodes or the average mastoid potential were selected as the reference electrode, it is demonstrated that the SNR of SSVEP is improved significantly as is the accuracy of SSVEP detection.

## Introduction

SSVEP-based BCI system possesses many advantages compared to other types of BCI system [1], [2], [3], [4], [5], [6], and one of the most prominent properties is its high transfer rate [7], [8], [9]. To get a high transfer rate, besides using a valid method for SSVEP extraction, it is most important to get an evoked EEG with a high signal to noise ratio (SNR). In an SSVEP-based BCI system, a widely used method for SSVEP extraction is to compute the SSVEP relative-power by FFT [4], [10], [11], and this method is referred to Power Spectrum (PS) Method. In fact, the relative-power of SSVEP can be seen as the SNR of SSVEP in a specific frequency band. In the Power Spectrum Method, a reference electrode (for example, ‘Cz’) is firstly selected, and then one or a few active electrodes (for example, ‘O1’ and/or ‘O2’) are selected [10], [12]. The relative-power of a certain frequency in spontaneous EEG at the active electrode is computed to build a threshold, and the relative-power of the corresponding frequency in evoked EEG at the active electrode is computed within a short period, such as 2 s or 3 s, then compared with the threshold. If the relative-power of that frequency is higher than the corresponding threshold, it can be concluded that this frequency SSVEP is included in this span, in other words, it can be detected that the subject is staring at the button with this frequency flicker inside and decides to select that button.

The BCI system which uses PS method is simple to build, because only one reference and one or a few active electrodes are employed [4], [8], [10], [13], [14], [15], [16]. However, because of the inter-subject difference of the SSVEP power [17], [18], [19], [20], [21], a certain active electrode may be very effective at a known frequency stimulus for a few subjects, while not so valid for the other subjects [22], [23], [24], [25]. Alternatively, even for the same subject, because of the traveling property of SSVEP [26], a certain active electrode may be very effective for a certain frequency stimulus while not so effective for the other frequency stimuli. In other words, the most effective active electrode can vary between subjects or different stimulus frequencies. To statically select only one or a few active electrodes, we cannot cover all of the most effective active electrodes, and thus must limit improvements to the SNR of SSVEP for all frequency stimuli.

Consider inter-subject differences and the traveling property of SSVEP. If all the electrodes on the scalp are selected as the active electrodes and the relative-power of SSVEP at these electrodes are summed together as an SSVEP indicator, the drawback of only utilizing one or a few active electrodes can be overcome to some extent. Although different frequency SSVEPs can come to their maximum power at different electrodes, these maximum powers can all be included in the sum relative-power. The signal at each electrode can make contributions to the recognition of SSVEP frequency, so the detection accuracy can be improved for all stimuli.

For a certain frequency SSVEP, the sum relative-power of SSVEP can vary with different reference electrodes [17]. In order to get the maximum sum relative-power, a suitable reference electrode should be confirmed dynamically for each frequency, and this reference electrode is referred to the optimum reference. The selection of the optimum reference electrode is conducted for every stimulus automatically. The optimum reference electrode can vary between subjects and different stimulus frequencies. The sum relative-power under the optimum reference is used as an indicator of SSVEP. The method of dynamically selecting an optimum reference electrode under the situation of selecting all electrodes as active electrodes is proposed for the first time in this work, and is referred to as the Dynamic Selection (DS) Method.

In this study, six frequencies in different bands were selected as the stimulus frequency, a 129 channel EEG system was used to record EEG signals, and 100 s length spontaneous EEG and evoked EEG were collected separately and then divided into 2 s length segments. For the evoked EEG segments, the sum relative-power of SSVEP for each frequency was computed according to the DS method, and compared to the sum relative-power under ‘Cz’ reference, the reference of the average potential at all electrodes, or the reference of the average mastoid potential, respectively. The results indicate that the sum relative-power under the optimum reference is significantly higher than the sum relative-power under other three kinds of reference; accordingly, the detection accuracy under the optimum reference is higher than that under the other three kinds of reference. Although the optimum reference electrodes for a certain frequency or a certain subject can be different from each other, most of them locate at the occipital lobe.

There are many other kinds of BCI system except for SSVEP-based BCI, such as P300-based BCI [27], sensorimotor rhythm (SMR)-based BCI [28]. The singal used for classfication in BCI can mainly be recorded over the scalp, over the cortical surface, and within the brain [29]. A new classification method of BCI which is simliar to the communication system is proposed recently [29], according to this method, the BCI system can be sorted into five types: TDMA, FDMA, CDMA, SDMA, and HMA. SSVEP-based BCI is the kind of FDMA, while P300-based BCI is the kind of TDMA. In these different kinds of BCI system, although the signal extraction methods are different, for example, FFT method is often used in SSVEP-based BCI, while the superposition method is often used in P300-based BCI, it is very important to get an evoked EEG with a high SNR. Although the method proposed in this study is based on the property of SSVEP, it can be extended to other type BCIs. For example, in a motor imagery based BCI, the ERP amplitude is related to the reference electrode. To select dynamically an optimum reference can lead to a high ERP amplitude, which can improve the classfication accuracy of the BCI system.

## Methodology

### 2.1 Ethics Statement

This study was approved by the Human Research and Ethics Committee of the University of Electronic Science and Technology of China. Before the experiment, all the subjects were told the purpose and procedure of the experiment in detail and signed a consent form. These forms were approved by the University of Electronic Science and Technology of China Ethics Committee Data Acquisition.

### 2.2 Data Acquirement

**Figure 1:**
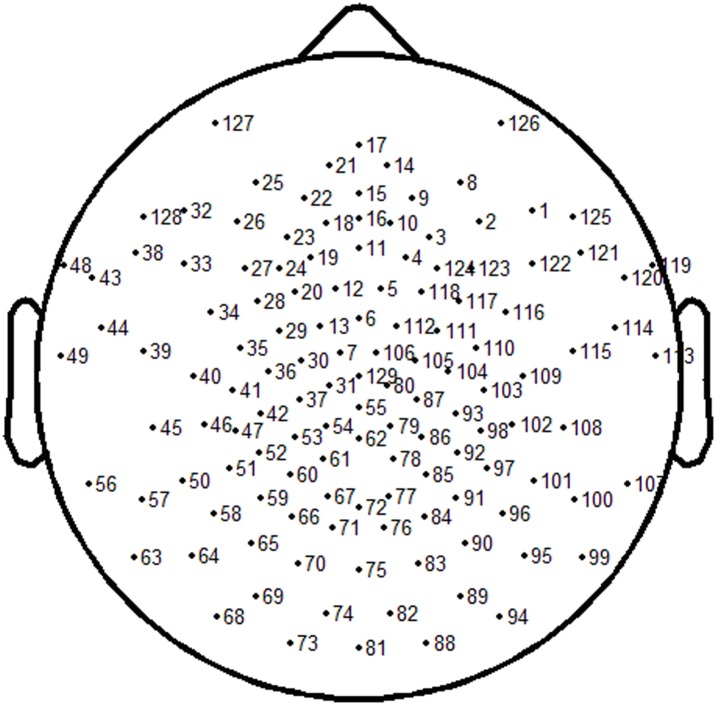
Electrodes location of 129 channel EEG system.

Eleven subjects were chosen to take part in this experiment, having either normal sight or corrected normal sight. Six subjects were male, and five subjects were female. The mean age was 24 (range 24±2) years old. The subjects were seated in a dark room, 60 cm from the stimulator. A high luminance focused LED was used as the SSVEP stimulator, driven by a pulse generator, and the duty-cycle of the pulse was set to 1∶1. The cycle of the pulse can only be adjusted in 1 ms step, and six cycles were selected, i.e. 30, 40, 60, 80, 120, and 160 ms, the corresponding six frequencies were 33.33, 25, 16.67, 12.5, 8.33, and 6.25 Hz, located at β, α, and θ band, respectively. This frequency arrangement can be used to study the validity of DS method for the fundamental frequency and the harmonics extraction. The order of these six stimuli was random for different subject, and this can cancel the probable influence resulted by the stimuli order. A 129-channel EEG system was used to collect the spontaneous EEG and evoked EEG. Electrode impedance was kept below 10 kΩ, and salt water dropped into the electrode periodically in order to retain good contact with the subjects scalp. Fig. 1 shows the location of the electrodes in this system. In the EEG recording stage, ‘Cz’ electrode (No. 129) was selected as the reference. In order to avoid power line interference, the cutoff frequency of the EEG system was set to 49 Hz. For each stimulus, 100 s length spontaneous EEG was collected first, and then 100 s length evoked EEG was collected. This spontaneous EEG was used to build the threshold for each frequency SSVEP.

### 2.3 Computation Method

Because the SSVEP has relative immunity to noise such as eye or body movement [2], [18], no pre-process method, such as removing eye movement was adopted in this work.

#### 2.3.1 SSVEP Gain Under Different Kinds of Reference

The original EEG was referenced at ‘Cz’ electrode. For 100 s length spontaneous EEG, FFT was applied directly, then a spectrum was attained at each electrode with a frequency resolution of 0.01 Hz. For a specific frequency ‘f’ Hz and the electrode L_1_, L_2_, … L_n_, the relative-power of ‘f’ Hz at any electrode ‘m’ can be computed as follows:
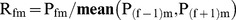
(1)Where ‘m’ is from ‘1’ to ‘n’, ‘R_fm_’ stands for the relative-power of ‘f’ Hz at electrode ‘m’, ‘P_fm_’ stands for the absolute-power of ‘f’ Hz at electrode ‘m’, ‘**mean**(P_(f–1)m_, P_(f+1)m_)’ stands for the average absolute-power from ‘f–1’ Hz to ‘f+1’ Hz at electrode ‘m’. The sum relative-power of ‘f’ Hz can be stated as:
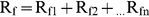
(2)Where ‘R_f_’ stands for the sum relative-power of ‘f’ Hz at all electrodes in spontaneous EEG, this sum relative-power is looked as SNR of this frequency in spontaneous EEG, and it is used as the baseline of this frequency. In this work, ‘n’ equals to 129, and ‘f’ equals to 33.33, 25, 16.67, 12.5, 8.33, and 6.25 Hz, respectively.

For evoked EEG, the same method as that for spontaneous EEG was adopted, and the sum relative-power of SSVEP was obtained which is looked as SNR of this frequency in evoked EEG. This sum relative-power of SSVEP was divided by the corresponding baseline in spontaneous EEG and the quotient referred to as the SSVEP gain under ‘Cz’ reference:
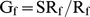
(3)Where ‘G_f_’ stands for SSVEP gain at ‘f’ Hz, ‘SR_f_’ stands for the sum relative-power of SSVEP at ‘f’ Hz in the evoked EEG, i.e. the SNR of SSVEP in evoked EEG, and the computation of ‘SR_f_’ in evoked EEG is the same as that of ‘R_f_’ in spontaneous EEG. This SSVEP gain under ‘Cz’ reference is compared to the next computed SSVEP gain under the optimum reference.

To compute the SSVEP gain under the other kinds of reference, the spontaneous EEG and evoked EEG at each electode were firstly re-refrenced at the average potential at all electrodes and the average potential at both mastoids, respectively. The average potential at electrode No. 56 and 107 was viewed as the average mastoid potential. The computation of SSVEP gain under these two kinds of references is the same as that under the ‘Cz’ reference.

#### 2.3.2 Selecting the Optimum Reference

For 100 s length evoked EEG of a known frequency, firstly the channel No. 1 is selected as the reference and the original signal at each electrode re-computed under this reference to get a new evoked EEG. FFT is applied on the new evoked EEG. The sum relative-power of SSVEP is computed using the same method as in 2.3.1. Following this, channel No. 2 is selected as the reference, and the same method as above applied. These steps are repeated until all channels have been selected as the reference once.

For the 129 channel EEG system, a total of 129 sum relative-power is computed and the maximum within them chosen as the optimum sum relative-power of SSVEP. The corresponding electrode is then selected as the optimum reference for the known frequency.

#### 2.3.3 SSVEP Gain Under the Optimum Reference

For spontaneous EEG, the sum relative-power of each stimulus frequency is re-computed under the corresponding optimum reference, and viewed as the baseline of each frequency under the optimum reference.

For evoked EEG, the sum relative-power of stimulus frequency is computed under the optimum reference, and this sum relative-power is divided by the corresponding baseline to get SSVEP gain under the optimum reference. The sum relative-power of the other five frequencies, which can be viewed as noise under the known stimulus, are computed under the corresponding optimum reference also, and divided by the corresponding baseline under the optimum reference to get noise gain. Noise gain is used to evaluate, while improving the SSVEP gain significantly under the optimum reference, whether the noise gain had improved appreciably or not.

#### 2.3.4 Detection Accuracy Under Different Kinds of Reference

Spontaneous EEG and evoked EEG were divided into segments of 2 s length separately. For each segment, ‘0’ series were added with a length of 2 s to get a whole 4 s length segment. The technique of adding ‘0’ series is utilized to improve the frequency resolution of FFT, and is widely applied in BCI studies [10], [11], [30]. In this work, if applying FFT directly on the 2 s length signal, the frequency resolution is 0.5 Hz. After appending ‘0’ series, the frequency resolution can be improved to 0.25 Hz. Then the four kinds of reference, i.e. ‘Cz’ reference, the reference of the average potential at all electrodes, the reference of average potential at mastoid, and the optimum reference, are applied to this 4 s length segment respectively to compute the sum relative-power. The sum relative-powers extracted from each spontaneous EEG segment is used to build a threshold for each frequency. An estimated threshold is used to check the sum relative-power of a certain frequency in spontaneous EEG segments, and adjusted continually until the detection accuracy equaled 90%. This means that the relative-power in 90% spontaneous EEG segments is smaller than this threshold and the adjusted value can be seen as the threshold of the corresponding frequency under a kind of reference. As a result, six thresholds can be confirmed under each type reference, respectively.

Under different kinds of reference, the sum relative-power in evoked EEG segments is compared to the corresponding threshold to check whether the SSVEP is included. First, only the first harmonic is utilized for detecting SSVEP with the checking standard being as follows: For an evoked EEG segment including a known frequency SSVEP, if the sum relative-power of the first harmonic exceeded its threshold, while the sum relative-powers of other frequencies except the second harmonic are all below the corresponding thresholds, the detection for this segment is correct. Secondly, for the high frequency stimuli such as 33.33 and 25 Hz, only the first harmonic is used for detecting, while for other middle and low frequency stimuli, both the first and second harmonics are utilized for detecting SSVEP. The checking standard used was as follows: For an evoked EEG segment including a known frequency SSVEP, if the sum relative-power of the first harmonic or the second harmonic exceeded the corresponding threshold, while the sum relative-powers of other frequencies are all below the corresponding thresholds, then the detection for this segment is correct.

For the results obtained via these steps, in order to test the significance of difference between methods, one-way Analysis of Variance (ANOVA) was applied. Significance level ‘p’ was selected as 0.05. If ‘p’ is smaller than 0.05, it suggests that there is a significant difference between the compared situations.

## Results

### 3.1 Optimum Reference Distribution

For the total 11 subjects, everyone took 6 SSVEP-frequency tests, so there were.

**Figure 2:**
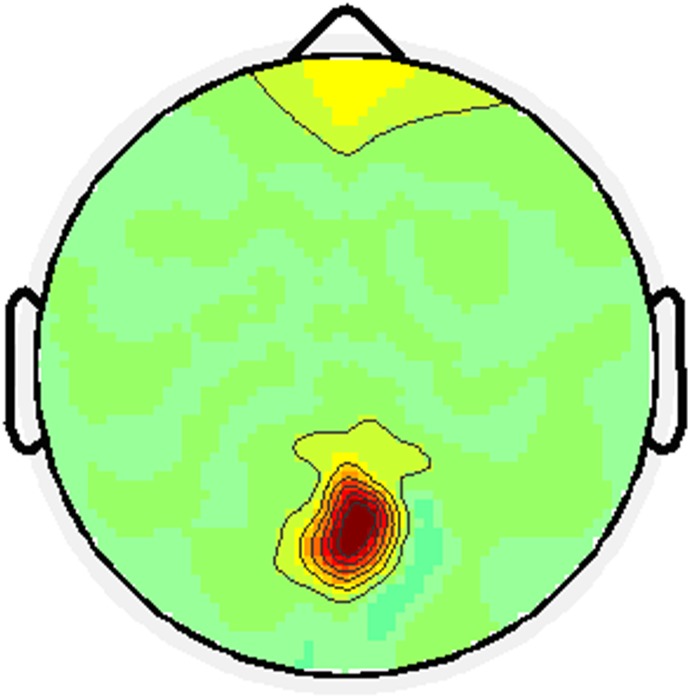
Optimum reference distribution topography. The deep color means more reference electrodes located in this area.

66 optimum reference electrodes chosen and most of these electrodes were located at the occipital area. For a certain subject, the optimum reference for different frequencies can be different. For a certain stimulus frequency, the optimum reference for different subjects can vary. Table 1 shows the optimum reference for different subjects at different stimuli. Fig. 2 shows the optimum reference distribution topography for all subjects under all stimuli.

**Table 1:** The optimum reference for different subject under diferent stimuli.

Subject	S1	S2	S3	S4	S5	S6	S7	S8	S9	S10	S11
Stimulus frequency (Hz)											
33.33	76	72	85	76	77	76	75	70	61	78	123
25	76	75	75	71	67	85	55	72	76	75	77
16.67	72	74	91	62	76	76	75	77	71	61	75
12.5	72	76	76	70	71	75	69	60	76	72	76
8.33	71	75	100	72	75	75	72	17	79	66	75
6.25	72	76	96	78	83	76	72	17	75	75	126

### 3.2 SSVEP’s SNR Improvement Under the Optimum Reference

Under a 33.33 Hz stimulus, the SNR of SSVEP under the optimum reference is significantly higher than that under ‘Cz’ reference (F(1,20) = 4.45, p = 0.04), the average mastoid reference (F(1,20) = 6.8, p = 0.03) and the commom average reference (F(1,20) = 5.47, p = 0.03), while for the other 5 frequencies which can be looked at as noise, the ANOVA results ‘p’ are far bigger than 0.05. This suggests that there is no significant improvement of the SNR for noise under the optimum reference. The SSVEP’s SNR improvement under the optimum reference can be seen from the average SSVEP gain across all subjects. For the stimulus frequency 33.33 Hz, the SSVEP gain under the optimum reference is 1.65 times of that under ‘Cz’ reference, 1.57 times of that under the average mastoid reference, and 1.7 times of that under the common average reference, while for other noise frequencies, this ratio is 1.0 or so, which means that the noise power in evoked EEG is the same level as that in spontaneous EEG under the optimum reference.

Under a 25 Hz stimulus, the comparison results between the optimum reference and the other kinds of reference were similar to that under 33.33 Hz stimuli. Under other middle or low frequency stimuli, i.e 16.67, 12.5, 8.33, and 6.25 Hz, the comparison results between the optimum reference and other three kinds of reference were similar to that under 33.33 Hz stimulus except that of the second harmonic. When stimulated at middle or low frequency, sometimes the second harmonic becomes stronger than the first harmonic under ‘Cz’ reference, or the average mastoid reference, or the common average reference, and the SNR of the second harmonic can be improved significantly when utilizing the optimum reference. Table 2 lists the average sum relative-power (SNR) across all subjects and the ANOVA results under every stimulus.

**Table 2:** Average SNR across all subjects and ANOVA results under every stimulus.

Frequency (Hz)			33.33	25	16.67	12.5	8.33	6.25
Spontaneous EEG	SNR under Cz reference		216.3	208.9	211.7	209.8	201.5	211.4
	SNR under average mastoid reference		211.5	212.3	213.1	214.7	210.2	207.8
	SNR under common average reference		214.2	209.4	215.1	211.7	213.8	200.9
	SNR under optimum reference		212.4	213.6	209.2	215.6	210.8	203.9
Stimulus frequency 33.33 Hz	Cz reference	SNR	720.5	211.7	208.9	215.4	198.7	223.2
		SSVEP/noise gain	3.33	1.01	0.99	1.03	0.99	1.06
	mastoid reference	SNR	710.8	209.3	210.5	211.1	210.8	215.9
		SSVEP/noise gain	3.36	0.99	0.99	0.98	1	1.04
	common reference	SNR	726.4	214.2	207.8	211.9	204.3	217.6
		SSVEP/noise gain	3.39	1.02	0.97	0.98	0.96	1.08
	Optimum reference	SNR	1167	220.2	219.3	211.1	194.7	203.1
		SSVEP/noise gain	5.49	1.03	1.05	0.98	0.92	1
	ANOVA ‘p’ (optimum vs Cz)		0.04	0.82	0.42	0.18	0.65	0.54
	ANOVA ‘p’ (optimum vs mastoid)		0.03	0.57	0.69	0.32	0.48	0.59
	ANOVA ‘p’ (optimum vs common)		0.03	0.72	0.33	0.46	0.63	0.37
Stimulus frequency 25 Hz	Cz reference	SNR	201.2	615.6	211.4	216.3	200.8	203.6
		SSVEP/noise gain	0.93	2.95	1	1.03	1	0.96
	mastoid reference	SNR	210.8	633.1	216.9	208.7	209.8	211.4
		SSVEP/noise gain	1	2.98	1.02	0.97	1	1.02
	common reference	SNR	212.3	617.2	211.8	210.9	217.5	215.9
		SSVEP/noise gain	0.99	2.95	0.98	1	1.02	1.07
	Optimum reference	SNR	211.3	904.9	215.6	203.3	186.7	205.6
		SSVEP/noise gain	0.99	4.24	1.03	0.94	0.89	1.01
	ANOVA ‘p’ (optimum vs Cz)		0.73	0.02	0.55	0.67	0.48	0.56
	ANOVA ‘p’ (optimum vs mastoid)		0.46	0.03	0.27	0.44	0.59	0.34
	ANOVA ‘p’ (optimum vs common)		0.66	0.01	0.35	0.57	0.32	0.64
Stimulus frequency 16.67 Hz	Cz reference	SNR	377.6	207.8	597.6	203.7	210.5	211.3
		SSVEP/noise gain	1.75	0.99	2.82	0.97	1.04	1
	mastoid reference	SNR	389.5	211.7	603.8	210.7	211.3	208.3
		SSVEP/noise gain	1.84	1	2.83	0.98	1.01	1
	common reference	SNR	373.7	209.8	612.9	213.7	219.2	204.8
		SSVEP/noise gain	1.74	1	2.85	1.01	1.03	1.02
	Optimum reference	SNR	464.4	224.4	974.1	215.9	218.9	213.4
		SSVEP/noise gain	2.19	1.05	4.66	1	1.04	1.05
	ANOVA ‘p’ (optimum vs Cz)		0.06	0.47	0.01	0.74	0.66	0.28
	ANOVA ‘p’ (optimum vs mastoid)		0.05	0.35	0.01	0.47	0.52	0.63
	ANOVA ‘p’ (optimum vs common)		0.1	0.57	0.02	0.33	0.72	0.35
Stimulus frequency 12.5 Hz	Cz reference	SNR	210.9	497.8	208.5	995.6	223.4	205.6
		SSVEP/noise gain	0.98	2.38	0.98	4.75	1.11	0.97
	mastoid reference	SNR	215.7	512.3	213.5	1004.8	216.7	210.4
		SSVEP/noise gain	1.02	2.41	1	4.68	1.03	1.01
	common reference	SNR	216.9	503.5	215.7	987.4	215.6	214.3
		SSVEP/noise gain	1.01	2.4	1	4.66	1.01	1.07
	Optimum reference	SNR	208.8	682.1	212.7	1503	225.6	224.1
		SSVEP/noise gain	0.98	3.19	1.02	6.97	1.07	1.1
	ANOVA ‘p’ (optimum vs Cz)		0.77	0.06	0.41	0.01	0.63	0.45
	ANOVA ‘p’ (optimum vs mastoid)		0.56	0.04	0.52	0.01	0.28	0.67
	ANOVA ‘p’ (optimum vs common)		0.44	0.04	0.38	0.01	0.39	0.55
Stimulus frequency 8.33 Hz	Cz reference	SNR	223.5	256.7	602.5	207.5	561.8	210.3
		SSVEP/noise gain	1.03	1.23	2.85	0.99	2.79	0.99
	mastoid reference	SNR	215.6	237.8	595.6	210.8	593.5	211.9
		SSVEP/noise gain	1.02	1.12	2.79	0.98	2.82	1.02
	common reference	SNR	227.4	232.8	606.9	210.9	600.3	215.4
		SSVEP/noise gain	1.06	1.11	2.82	1	2.81	1.07
	Optimum reference	SNR	239.1	269.5	927.9	193.1	870.8	216.6
		SSVEP/noise gain	1.13	1.26	4.44	0.9	4.13	1.06
	ANOVA ‘p’ (optimum vs Cz)		0.65	0.42	0.03	0.57	0.02	0.49
	ANOVA ‘p’ (optimum vs mastoid)		0.47	0.33	0.02	0.39	0.01	0.66
	ANOVA ‘p’ (optimum vs common)		0.39	0.55	0.02	0.47	0.02	0.27
Stimulus frequency 6.25 Hz	Cz reference	SNR	209.7	225.9	211.4	747.4	209.8	523.8
		SSVEP/noise gain	0.97	1.08	1	3.56	1.04	2.48
	mastoid reference	SNR	214.4	209.5	217.8	699.8	213.2	540.3
		SSVEP/noise gain	1.01	0.99	1.02	3.26	0.99	2.6
	common reference	SNR	211.3	214.5	216.4	726.5	211.9	535.9
		SSVEP/noise gain	0.99	1.02	1.01	3.43	0.99	2.67
	Optimum reference	SNR	201.3	239.5	226.2	1129	214.1	890.5
		SSVEP/noise gain	0.95	1.12	1.08	5.23	1.02	4.38
	ANOVA ‘p’ (optimum vs Cz)		0.59	0.44	0.23	0.03	0.76	0.01
	ANOVA ‘p’ (optimum vs mastoid)		0.43	0.38	0.41	0.02	0.59	0.01
	ANOVA ‘p’ (optimum vs common)		0.63	0.27	0.56	0.02	0.51	0.01

### 3.3 Detection Accuracy Under Different Kinds of Reference

**Figure 3:**
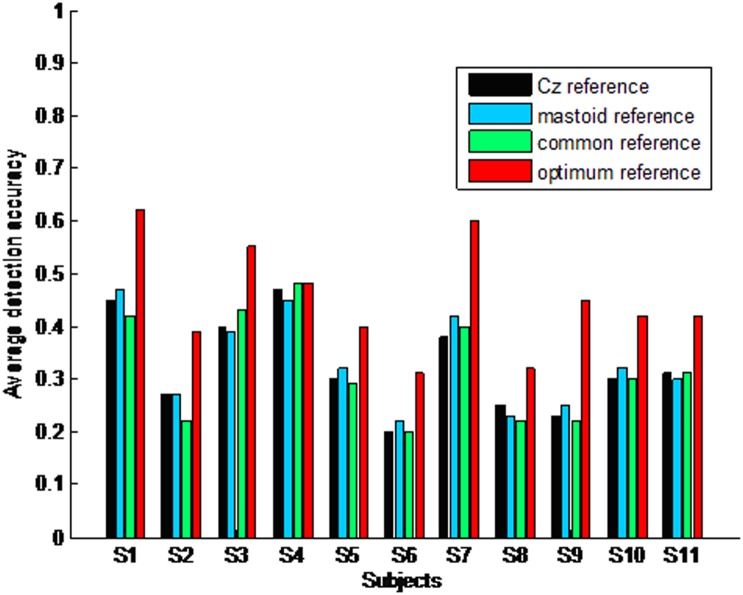
Average detection accuracy across all stimuli for every subject when only taking the first harmonic into account.

When only taking the first harmonic into account, the average detection accuracy across all subjects and stimuli is low under all kinds of references, i.e. 31.2% under ‘Cz’ reference, 34.4% under the average mastoid reference, 33.2% under the common average reference and 42.9% under the optimum reference. However, the average detection accuracy across all stimuli under the optimum reference is significantly higher than that under ‘Cz’ reference (F(1, 20) = 8.19, p = 0.01), the average mastoid reference (F(1, 20) = 6.3, p = 0.01), and the common average reference (F(1, 20) = 10.2, p = 0.01). For all subjects, the detection accuracy of different frequencies is different. Normally, the detection accuracy of middle and low frequencies such as 16.67, 12.5, 8.33, and 6.25 Hz is lower than that of high frequencies such as 33.33 and 25 Hz. Fig. 3 illustrates the average detection accuracy across all stimuli for every subject when only taking the first harmonic into account.

**Figure 4:**
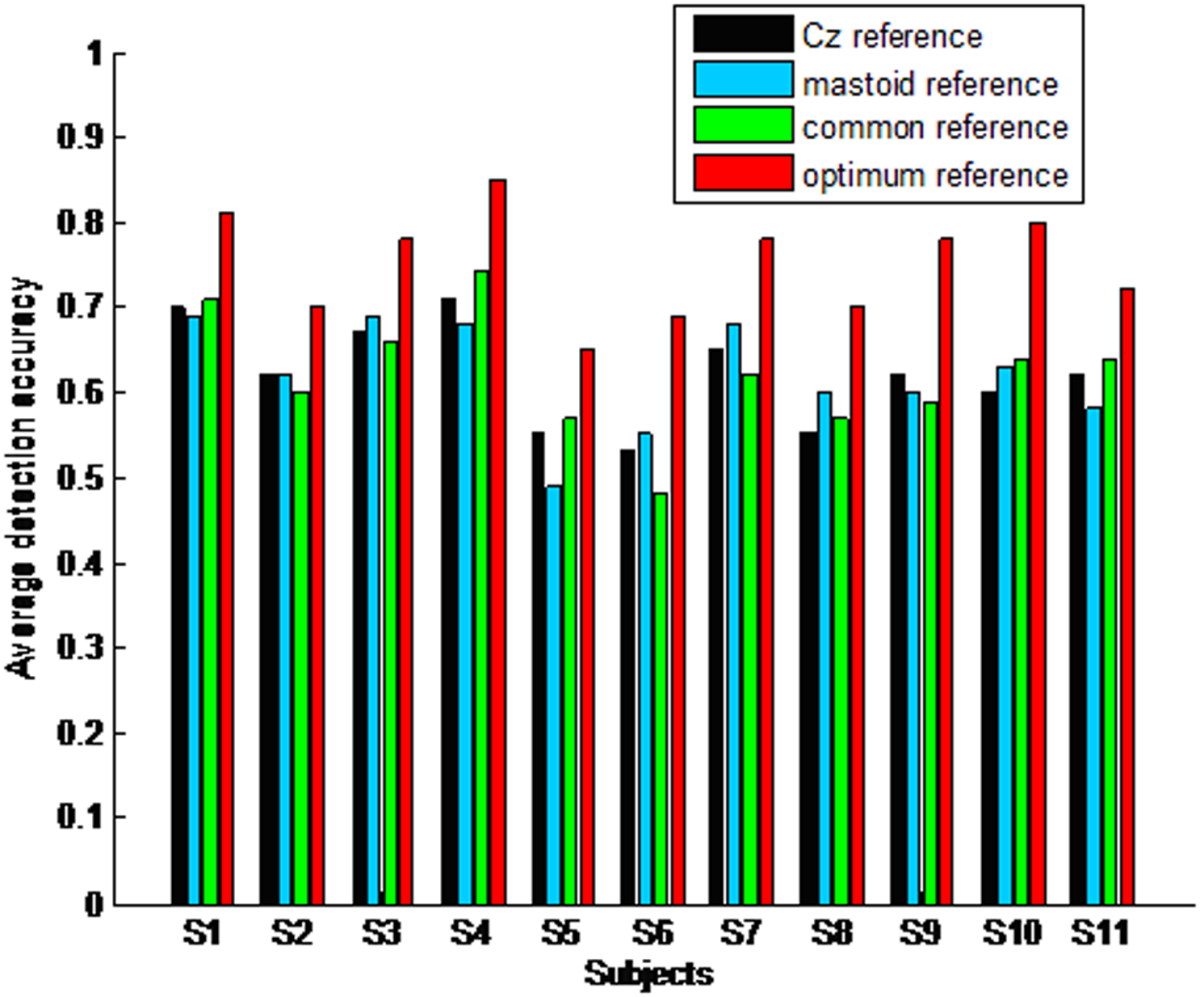
Average detection accuracy across all stimuli for every subject when taking the first and second harmonic into account.

When taking the first harmonic into account for high frequency stimuli such as 33.33 and 25 Hz (due to not recording the second harmonic), and taking the first and second harmonic into account for middle and low frequency stimuli, such as 16.67, 12.5, 8.33, and 6.25 Hz, the average detection accuracies across all stimuli can be improved significantly under all kinds of reference. Furthermore, the average detection accuracies across all subjects and stimuli are 62.2% under ‘Cz’ reference, 61.8% under the average mastoid, 61.6% under the common average and 73.7% under the optimum reference, respectively. In this situation, the average detection accuracy across all stimuli under the optimum reference is significantly higher than that under ‘Cz’ reference (F(1,20) = 11.3, p = 0.0), the average mastoid reference (F(1, 20) = 9.52, p = 0.0), and the common average (F(1, 20) = 8.3, p = 0.0). For all subjects, the detection accuracy of different frequencies is different. Normally, the detection accuracy of middle and low frequency stimuli such as 16.67, 12.5, 8.33, and 6.25 Hz is higher than that of high frequency stimuli such as 33.33 and 25 Hz. Fig. 4 illustrates the average detection accuracy across all stimuli for every subject when taking both the first and second harmonic into account.

Table 3 lists the average detection results across all subjects under different stimulus frequencies. From this table, it can be seen that when using middle or low frequency stimuli, the second harmonic is very important for SSVEP detection. Under any situation, the ANOVA ‘p’ is far smaller than 0.05, which suggests that the detection accuracy under optimum reference is significantly higher than that under other three kinds of reference.

**Table 3:** Average detection accuracy across all subjects under different situations.

Stimulus Frequency (Hz)		33.33	25	16.67	12.5	8.33	6.25
Detection using the first harmonic	Accuracy (Cz reference)	0.49	0.39	0.3	0.27	0.22	0.2
	Accuracy (mastoid average reference)	0.46	0.4	0.29	0.28	0.22	0.2
	Accuracy (common average reference)	0.47	0.4	0.31	0.27	0.23	0.21
	Accuracy (optimum reference)	0.63	0.5	0.44	0.42	0.27	0.32
	ANOVA ‘p’ (optimum vs Cz)	0.01	0.01	0.01	0.02	0.04	0.02
	ANOVA ‘p’ (optimum vs mastoid)	0.01	0.01	0.01	0.03	0.03	0.01
	ANOVA ‘p’ (optimum vs common)	0.01	0.02	0.01	0.01	0.02	0.01
Detection using the first and second harmonic	Accuracy (Cz reference)	0.49	0.39	0.7	0.68	0.72	0.75
	Accuracy (mastoid average reference)	0.46	0.4	0.71	0.7	0.71	0.75
	Accuracy (common average reference)	0.47	0.4	0.7	0.72	0.72	0.73
	Accuracy (optimum reference)	0.63	0.5	0.79	0.83	0.85	0.82
	ANOVA ‘p’ (optimum vs Cz)	0.0	0.0	0.01	0.0	0.0	0.01
	ANOVA ‘p’ (optimum vs mastoid)	0.01	0.01	0.01	0.0	0.0	0.01
	ANOVA ‘p’ (optimum vs common)	0.0	0.01	0.01	0.0	0.01	0.01

## Discussion

In this work, even in the consideration of the second harmonic, the detection accuracy for some subjects is still low compared to other works [10], [11], this is resulted by the experiment itself. SSVEP power is related to factors such as stimulus intensity, frequency, modulation depth and to the subjects themselves. This work shows that there is considerable intra-difference for the same subject between different frequency stimuli, and there is great inter-difference between subjects even under the same stimuli. These differences lead to the different detection accuracy ratios between subjects. The experiment in this work can lead to more inter-subject or intra-subject differences. Although every subject was asked to be seated in the same location, we have not confirmed whether the subject evoked a maximum SSVEP. Because a focused LED was used as the stimulator of SSVEP in this study, if the subject did not stare at the flicker from the correct vision angle, the light projecting into the eyes decreased acutely and the maximum SSVEP was not evoked. In fact, we have studied the spectrum of the 100 s length evoked EEG, in some trials, the peak is not clear, which suggests that the SSVEP is very weak. Therefore, detection accuracy in this work is not as high as that in the other real BCI experiments [10], [11]. However, this does not matter for the comparison between the methods for reference electrode selection. In a real SSVEP-based BCI, the experiment design is very important. Firstly, suitable frequency arrangements can lead to a high SSVEP power and reduce the interference between stimuli. For example, normally selecting a middle or low frequency can evoke a large SSVEP, and a certain stimulus frequency should be far enough from other stimuli and their harmonics. Secondly, stimulator selection is also very important. We have compared the influence on SSVEP power by different stimulators [31]. When using a CRT monitor or emanative LED, the subject can receive almost the same strength light in a wide range, so a little shift of the vision angle does not affect SSVEP power. However, when using a focused LED as the stimulator, the light concentrates in a narrow area, the shift of the vision angle has a great influence on SSVEP power. So, in a multi-target BCI system, because of the vision angle shifting significantly, the focused LED should be avoided.

In order to check the influence on the second harmonic by the dynamic selection method, the SNR of the second harmonic was also computed Firstly, only the first harmonic was used to detect SSVEP. Then the first and second harmonic were used together to detect SSVEP. The results show that, when stimulating at a middle or low frequency, the SNR of the second harmonic can be improved significantly when using the optimum reference, accordingly the detection accuracy using the first and second harmonic together is significantly higher than when only using the first harmonic. In order to avoid the power line interference of 50 Hz, the cutoff frequency of the recording system is set 49 Hz, so a high frequency harmonic of stimulus such as 25 and 33.33 Hz was not collected. In consequence, the detection accuracy for these stimuli is smaller than that of the low frequency stimuli. In an SSVEP-based BCI, the harmonics are very important for improving detection accuracy, so the first and second harmonics, sometimes even the third harmonic, should be taken into account even under the optimum reference. In fact, some works have discussed the usefulness of harmonics in detail [4], [10].

In past SSVEP-based BCI systems, often one or a few active electrodes were chosen from the electrodes located at the occipital area [1], [10], [11], [13], [32]. There are some drawbacks to doing this. For the same frequency, the optimum active electrode in the occipital area may be different because of inter-subject differences. In order to get the highest detection accuracy for each subject, it should be tested first to confirm the optimum active electrode before detecting targets. This however, is a waste of manpower and time [11], [24], [33]. Although the occipital area is considered the source of SSVEP [25], suggesting SSVEPs in this area are normally higher than those in other areas, for different frequencies, the location of the maximum SSVEP in the occipital area is different. In a multi-targets SSVEP-based BCI system, there are normally many frequencies applied, it is impossible to find an electrode in the occipital area which is optimum for all frequencies. So, all the electrodes are selected as the active electrode in this work. Under this selection, for any subject and any stimulus frequency, there is no need to confirm the optimum active electrode before detection. The SSVEP power in different areas can all be collected as an indicator of SSVEP, and this indicator includes more information than that for only one or a few active electrodes selected. Therefore, the detection accuracy for all stimuli is improved significantly.

When selecting all electrodes as the active electrode, it is important to find an optimum reference under which the sum relative-power of SSVEP is at a maximum. Under the optimum reference, the SNR of SSVEP is improved significantly compared to the other kinds of reference. While using the optimum reference to improve the SSVEP gain, if the noise gain is improved the same level as SSVEP gain, optimum reference makes no improvement for SSVEP detection accuracy. Fortunately, except for the second harmonic, the other noise relative-power is not increased under optimum reference. Consequentially, when using the first and second harmonic together to detect SSVEP under the optimum reference, a higher detection accuracy compared to the other kinds of reference can be attained.

Although the optimum references for different subjects or different stimulus frequencies can differ, most of them are located at the occipital area. This can be understood through the following analysis. The SSVEP is a response to the visual stimulus mainly by the primary visual cortex, and the occipital area is the source of SSVEP. Here we hypothesize an ideal situation, i.e. there is an ideal reference, which is not related to EEG at any electrode, and only one electrode ‘E_0_’ at the occipital area is the SSVEP source. Furthermore, the SSVEP at electrodes ‘E_1_’,… ‘E_m_’… ‘E_n_’ are transferred from ‘E_0_’ via the scalp. The SSVEP between the ideal reference and electrode ‘E_0_’, ‘E_1_’,… ‘E_m_’… ‘E_n_’ are expressed:
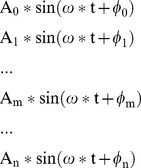
(4)


A_0_, A_1_,.A_m_…A_n_ are the SSVEP amplitude at different electrodes compared to the ideal reference, ‘ω’ is the SSVEP frequency, Φ_0_, Φ_1_, …Φ_m_… Φ_n_ are the initial phase of SSVEP at different electrodes. Because of the travelling property of SSVEP and the attenuation characteristics of the scalp, the amplitude A_0_, A_1_, … A_m_, … A_n_ can be different and A_0_ is the biggest among these amplitudes, the initial Φ_0_, Φ_1_, … Φ_m_… Φ_n_ can be different also.

When using electrode ‘E_0_’ as the reference, the relative SSVEP at other electrodes ‘E_1_’,… ‘E_m_’… ‘E_n_’ can be stood by:
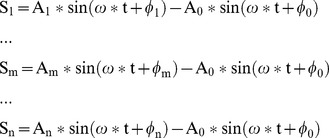
(5)


And the sum power ‘P_0_’ of these signals is:
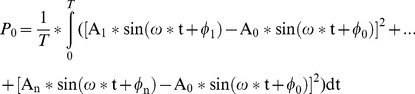
(6)Where ‘T’ is the cycle of the frequency ‘ω’.

When using electrode ‘E_m_’ as the reference, the relative SSVEP at other electrodes is represented:
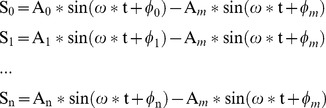
(7)


And the sum power ‘P_m_’ of these signals is.
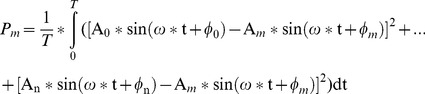
(8)


The ‘P_0_’ and ‘P_m_’ can be computed, and the difference of the sum power under reference ‘E_0_’ and ‘E_m_’ is illustrated as:
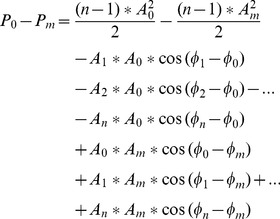
(9)


This difference is related to SSVEP amplitude and initial phase at every electrode. The different attenuation properties of a subject’s scalp can lead to a different distribution of SSVEP. Even for the same subject, the distribution of SSVEP amplitude and initial phase can be different for different stimulus frequencies. It is impossible to prove ‘P_0_-P_m_’ is bigger or smaller than zero using mathematical methods. In order to understand this, a group data including 129 amplitudes and 129 initial phases are simulated randomly 100,000 times in a computer to compute equation (9). Except for assuming the source electrode ‘E_0_’ with the maximum amplitude, there is no other limitation for other amplitudes and all initial phases, this suggests these simulant data can include all the real SSVEP amplitude and initial phase. The results show that the difference ‘P_0_-P_m_’ is bigger than zero in more than an average probability of 98%. In other words, even using the real amplitudes and initial phases in place of those in equation (9), ‘P_0_’ is bigger than ‘P_m_’ in most situations. This suggests that, if using SSVEP source as the reference, a maximum sum relative-power can be attained. Therefore, in the dynamic selection method, most optimum references locate at the occipital area.

There is a very great difference between the method proposed in this work and the method in which the average potential at all electrodes in the time domain are selected as the reference. When selecting the average potential at all electrodes in the time domain as a reference, although the background noise can be canceled to some extent, more amplitude information of SSVEP has been canceled on average because of the traveling features of SSVEP, and thus cannot lead to the maximum sum relative-power of SSVEP. In this work, the sum relative-power at all electrodes in the frequency domain is selected as the indicator of SSVEP. The phase of SSVEP has not been taken into account, and the SSVEP amplitude information at all electrodes remains in the sum relative-power. Maximum sum relative-power of SSVEP can be identified.

The cost of the dynamic selection method is the increased complexity of the system because more electrodes can be fixed. Except for this drawback, the procedures using the dynamic selection method are easy to apply. During the stage of building a threshold for each stimulus, the spontaneous EEG within a time period is first obtained, then each stimulus frequency is used once to evoke SSVEP for a period of length the same as for the spontaneous EEG. Ultimately, the optimum reference for each stimulus is confirmed automatically, and the threshold under this optimum reference is computed automatically also. Therefore, time consumption in this stage is minimal. Alternatively, with the other reference selection method, normally finding a suitable active electrode for all stimuli and confirming the threshold is very time consuming. In the formal detection stage, the procedure is similar to that of the power spectrum method, i.e. the power of every adopted frequency under the corresponding optimum reference is computed and compared to the corresponding threshold. If every power is smaller than its corresponding threshold, then there is no button selected. If there is only one power bigger than its corresponding threshold, the button with the corresponding frequency flicker inside is selected. If there are more powers bigger than their corresponding thresholds, other techniques are applied to confirm which button is valid, for example, taking the results as invalid, or selecting the frequency, which has the highest SSVEP gain as the target frequency.

Too many electrodes adopted in the DS method can limit the popularization of this method. In order to understand the influences of the number of electrodes for the detection accuracy, we reduce the electrode density, for example, only selecting one third electrodes in each lobe, the results show the optimum reference concentrates mostly at the occipital lobe. Although the detection accuracy under sitution of lower intensity electrode is sometimes a little lower than that under all electrodes, compared to the other three kinds of reference, the detection accuracy of DS method is still the highest. In consideration of the optimum reference concentrating mostly at the occipital lobe, in order to decrease the complexity of BCI system, we can use only the electrodes in occipital area for EEG recording, and select the optimum reference from these electrodes for SSVEP detection.

## Conclusion

Compared to other SSVEP extraction methods, in which one reference is selected statically and one or a few electrodes in the occipital area are chosen as the active electrode, Dynamic Selection Method uses more active electrodes, and thus can increase the complexity of the system. The method of Dynamic Selection Method improves SSVEP’s SNR and detection accuracy significantly and is easy to employ by decreasing the number of electrodes, thus being applicable in a real time SSVEP-based BCI.

## References

[1] Luo A, Sullivan TJ (2010) A User-friendly SSVEP-based brain–computer Interface Using a Time-domain Classifier. J. Neural Eng. 7: 1–10. 10.1088/1741-2560/7/2/026010 20332551

[2] Allison BZ, Wolpaw EW, Wolpaw JR (2007) Brain-computer Interface Systems: Progress and Prospects. Expert Review of Medical Devices 4(No (4)) 463–474. 17605682 10.1586/17434440.4.4.463

[3] Vialatte FB, Maurice M, Dauwels J, Cichocki A (2010) Steady-state Visually Evoked Potentials: Focus on Essential Paradigms and Future Perspectives. Progress in Neurobiology 90: 418–438. 19963032 10.1016/j.pneurobio.2009.11.005

[4] Muller-Putz GR, Scherer R, Brauneis C, Pfurtscheller G (2005) Steady-state Visual Evoked Potential (SSVEP)-based Communication: Impact of Harmonic Frequency Components. J. Neural Eng. 2: 123–130. 10.1088/1741-2560/2/4/008 16317236

[5] Ghaleb I, Davila CE, Srebro R (1996) Detection of Near Threshold Contrast Visual Evoked Potentials Using Coherent Detection Techniques. Biomedical Engineering Conference 121–124.

[6] Wolpaw JR, Birbaumer N, McFarland DJ, Pfurtscheller G, Vaughan TM (2002) Brain-computer Interfaces for Communication and Control. Clinical Neurophysiology 113: 767–791. 12048038 10.1016/s1388-2457(02)00057-3

[7] Lopez MA, Pelayo F, Madrid E, Alberto P (2009) Statistical Characterization of Steady-State Visual Evoked Potentials and Their Use in Brain–Computer Interfaces. Neural Process Lett 29: 179–187.

[8] Middendorf M, Mcmillan G, Calhoun G, Jones KS (2008) Brain-Computer Interfaces Based on the Steady-state Visual-Evoked Response. IEEE Transactions on Rehabilitation Engineering 8(No. 2): 211–214. 10.1109/86.847819 10896190

[9] Sami S, Nielsen KD (2004) Communication speed enhancement for visual based Brain Computer Interfaces 9th Annual Conference of the International FES Society 475–480.

[10] Cheng M, Gao XR, Gao SK, Xu DF (2002) Design and Implementation of a Brain-Computer Interface with High Transfer Rates. IEEE Trans. BME VOL. 49(No (10)) 1181–1186. 10.1109/tbme.2002.803536 12374343

[11] Gao XR, Xu DF, Cheng M, Gao SK (2003) A BCI Based Environmental Controller for the Motion-Disabled. IEEE Transactions on Neural System and Rehabilitation Engineering 11(No (2)) 137–140. 10.1109/TNSRE.2003.814449 12899256

[12] Hwang HJ, Lim JH, Jung YJ, Choi H, Lee SW, et al (2012) Development of an SSVEP-based BCI spelling system adopting a QWERTY-style LED keyboard. Journal of Neuroscience Methods 208: 59–65. 22580222 10.1016/j.jneumeth.2012.04.011

[13] Lopez MA, Praetor A, Playa F (2010) Use of Phase in Brain–Computer Interfaces Based on Steady-State Visual Evoked Potentials. Morillas Neural Process Lett 32: 1–9.

[14] Molina GG, Tsoneva T, Nijholt A (2013) Emotional brain–computer interfaces. Int. J. Autonomous and Adaptive Communications Systems 6: 1–9.

[15] Rosario AO, Adeli H (2013) Brain-computer interface technologies: from signal to action. Reviews in the Neurosciences 24: 455–562. 24077619 10.1515/revneuro-2013-0032

[16] McCullagh P, Galway L, Lightbody G (2013) Investigation into a Mixed Hybrid Using SSVEP and Eye Gaze for Optimising User Interaction within a Virtual Environment. Lecture Notes in Computer Science 8009: 530–539.

[17] Herrmann CS (2001) Human EEG Responses to 1–100 Hz Flicker: Resonance Phenomena in Visual Cortex and Their Potential Correlation to Cognitive Phenomena. Exp Brain Res (137) 346–353. 10.1007/s002210100682 11355381

[18] Carlos ED, Alireza A, Alireza K (1994) Estimation of Single Sweep Steady-State Visual Evoked Potentials by Adaptive Line Enhancement. IEEE Trans. BME 41(No. 2): 197–200. 10.1109/10.284933 8026854

[19] Cheng M, Gao XR, Gao SK, Wang BL (2005) Stimulation Frequency Extraction in SSVEP-based Brain-computer Interface. 2005 First International Conference on Neural interface and Control Proceeding 64–67.

[20] Kelly SP, Lalor EC, Reilly RB, Foxe JJ (2005) Visual Spatial Attention Tracking Using High-Density SSVEP Data for Independent Brain-Computer Communication. IEEE Transactions on Neural System and Rehabilitation Engineering 13(No. 2): 172–178. 10.1109/TNSRE.2005.847369 16003896

[21] Lin ZL, Zhang CS, Wu W, Gao XR (2007) Frequency Recognition Based on Canonical Correlation Analysis for SSVEP-Based BCIs. IEEE Trans. BME 54(No. 6): 1172–1176. 10.1109/tbme.2006.889197 17549911

[22] Birbaumer N, Cohen LG (2007) Brain-computer Interfaces: Communication and Restoration of Movement in Paralysis. The Journal of Physiology 579: 621–636. 17234696 10.1113/jphysiol.2006.125633 PMC2151357

[23] Lee PL, Sie JJ, Liu YJ (2010) An SSVEP-Actuated Brain Computer Interface Using Phase-Tagged Flickering Sequences: A Cursor System. Annals of Biomedical Engineering 38: 2383–2397. 20177780 10.1007/s10439-010-9964-y

[24] Wang RP, Zhang ZG, Gao XR (2005) Electrode Selection for SSVEP-based Binocular Rivalry. 2005 First International Conference on Neural Interface and Control Proceeding 75–78.

[25] Wang YJ, Zhang ZG, Gao XR (2004) Electrode Selection for SSVEP-based Brain-computer Interface. Proceeding of the 26th Annual International Conference of the IEEE EMBS 4507–4510. 10.1109/IEMBS.2004.1404252 17271308

[26] Burkitt GR, Silberstein RB, Cadusch PJ, Wood AW (2000) Steady-state Visual Evoked Potentials and Travelling Waves. Clinical Neurophysiology 111: 246–258. 10680559 10.1016/s1388-2457(99)00194-7

[27] Yin E, Zhou Z, Jiang J, Chen FL, Liu YD, et al (2013) A novel hybrid BCI speller based on the incorporation of SSVEP into the P300 paradigm. Journal of Neural Engineering 10: 1–10. 10.1088/1741-2560/10/2/026012 23429035

[28] Yan H, He B (2014) Brain-Computer Interfaces Using Sensorimotor Rhythms: Current State and Future Perspectives. BME VOL.61(No. 5): 1425–1435. 10.1109/TBME.2014.2312397 PMC4082720 24759276

[29] Gao SK, Wang YJ, Gao XR, Hong B (2014) Visual and Auditory Brain-Computer Interfaces. IEEE Trans. BME VOL.61(No. 5): 1436–1447. 10.1109/TBME.2014.2300164 24759277

[30] Wu ZH, Yao DZ (2008) Frequency detection with stability coefficient for SSVEP based BCIs. J. Neural Eng. 5: 36–43. 10.1088/1741-2560/5/1/004 18310809

[31] Wu ZH, Lai YX, Xia Y, Wu D, Yao DZ (2008) Stimulator selection in SSVEP-based BCI. Medical Engineering & Physics 30: 1079–1088. 18316226 10.1016/j.medengphy.2008.01.004

[32] Friman O, Volosyak I, Graser A (2007) Multiple Channel Detection of Steady-State Visual Evoked Potentials for Brain-Computer Interfaces. IEEE Trans. BME 54(No. 4): 742–750. 10.1109/TBME.2006.889160 17405382

[33] Ridder WH, McCulloch D, Herbert AM (1998) Stimulus Duration, Neural Adaptation, and Sweep Visual Evoked Potential Acuity Estimates. Invest Ophthalmol. Vis. Sci. 39: 2759–2768. 9856787

